# Sonic Hedgehog Controls the Phenotypic Fate and Therapeutic Efficacy of Grafted Neural Precursor Cells in a Model of Nigrostriatal Neurodegeneration

**DOI:** 10.1371/journal.pone.0137136

**Published:** 2015-09-04

**Authors:** Lalitha Madhavan, Brian F. Daley, Beverly L. Davidson, Ryan L. Boudreau, Jack W. Lipton, Allyson Cole-Strauss, Kathy Steece-Collier, Timothy J. Collier

**Affiliations:** 1 Department of Neurology, University of Arizona, Tucson, Arizona, 85724, United States of America; 2 Center for Cell and Molecular Therapy, The Children’s Hospital of Philadelphia, Philadelphia, Pennsylvania, 19104, United States of America; 3 Department of Internal Medicine, University of Iowa, Iowa City, Iowa, 52242, United States of America; 4 Translational Science and Molecular Medicine, Michigan State University, Grand Rapids, Michigan, 49503, United States of America; University of South Florida, UNITED STATES

## Abstract

The expression of soluble growth and survival promoting factors by neural precursor cells (NPCs) is suggested to be a prominent mechanism underlying the protective and regenerative effects of these cells after transplantation. Nevertheless, how and to what extent specific NPC-expressed factors contribute to therapeutic effects is not well understood. Using RNA silencing, the current study investigated the roles of two donor NPC molecules, namely glial cell-line derived neurotrophic factor (GDNF) and sonic hedgehog (SHH), in the protection of substantia nigra dopamine neurons in rats treated with 6-hydroxydopamine (6-OHDA). Analyses indicate that as opposed to the knock-down of GDNF, SHH inhibition caused a profound decline in nigrostriatal neuroprotection. Further, SHH silencing also curbed endogenous neurogenesis and the migration of host brdU^+^/dcx^+^ neural precursors into the striatum, which was present in the animals receiving control or GDNF silenced NPCs. A change in graft phenotype, mainly reflected by a reduced proportion of undifferentiated nestin^+^ cells, as well as a significantly greater host microglial activity, suggested an important role for these processes in the attenuation of neuroprotection and neurogenesis upon SHH silencing. Overall these studies reveal core mechanisms fundamental to grafted NPC-based therapeutic effects, and delineate the particular contributions of two graft-expressed molecules, SHH and GDNF, in mediating midbrain dopamine neuron protection, and host plasticity after NPC transplantation.

## Introduction

Neural precursor cells (NPCs) can spontaneously express a diverse array of diffusible factors upon transplantation into brain tissues. Through such paracrine abilities, grafted NPCs can support the robust survival and function of surrounding neural cells, and induce plasticity and repair in the recipient brain [1–8]. However, whether such NPC-derived factors play causal roles in therapeutic effects observed after transplantation, and the extent and nature of involvement of specific donor cell expressed factors, remains an active area of investigation.

In the current work, we investigate the participation of two grafted NPC expressed factors in mediating nigrostriatal neuroprotection in a 6-OHDA rat model of Parkinson’s disease (PD). Our previous studies have established that postnatal NPCs, obtained from the subventricular zone (SVZ) of newborn rats, induce neuroprotection when grafted prior to a 6-OHDA toxin insult, and that this phenomenon is associated with the immunocytochemical expression of specific factors in the grafted cells, including glial cell line-derived neurotrophic factor (GDNF) and sonic hedgehog (SHH) [4, 9] [5]. Given this, we examined the potential involvement of these specific donor NPC-derived factors in the observed neuroprotection, using targeted RNA interference (RNAi) methods. In particular, short hairpin lentiviral RNAi was used to silence expression of SHH, GDNF, or both, in the NPCs *in vitro*. These genetically silenced NPCs were subsequently transplanted into the striatum and substantia nigra (SN) of adult Fisher 344 rats, which were later treated with 6-OHDA infused into the ipsilateral median forebrain bundle to induce nigrostriatal degeneration. Behavioral and histological analyses indicate that the decreased expression of SHH (but not GDNF) negatively affects the ability of grafted NPCs to induce host neurogenesis, beneficially modulate host glial activity, and protect midbrain dopamine (DA) neurons. To our knowledge, this is first evidence that clearly defines the influences of specific NPC-derived factors in governing the nature of graft-host interactions and host neuroprotection after transplantation.

## Materials and Methods

### NPC culture

NPCs were isolated from SVZs of newborn transgenic Fischer 344 rats, expressing human placental alkaline phosphatase (hPAP), using previously established methods [5]. The expression of hPAP allows identification of grafted NPCs, labeled with this human marker, within the brains of wild-type host rats [10, 11]. The NPCs were grown in uncoated dishes and serum-free neurobasal-A medium *(Invitrogen*, *Carlsbad*, *CA)* supplemented with 2% B27 *(Invitrogen*, *Carlsbad*, *CA)*, 20 ng/ml epidermal growth factor (EGF, *Cell Sciences*, *Canton*, *MA*), 10 ng/ml basic fibroblast growth factor (bFGF, *Cell Sciences*, *Canton*, *MA*), 5 ug/ml heparin *(Stemcell Technologies*, *Vancouver*, *BC*, *Canada)*, 500 μM glutamax, 200 units/ml penicillin, and 200 μg/ml streptomycin (*Invitrogen*, *Carlsbad*, *CA)*, at 37°C [5].

### Quantitative real time polymerase chain reaction (qRT-PCR)

qRT-PCR was conducted as described [12]. Briefly, RNA was isolated from NPCs using the RNeasy Plus Mini kit (Qiagen, Valencia, CA), after which it was converted to cDNA via a two-step process using random hexamers and MultiScribe Reverse Transcriptase (ABI, Carlsbad, CA). The PCR reactions were carried out with 1X TaqMan Universal MasterMix (ABI, Carlsbad, CA) and GDNF or SHH primer probes. The qPCR reactions were run on an ABI 7500 real-time thermo-cycler, and reactions to control for cDNA quantities were run using glyceraldehyde 3-phosphate dehydrogenase (GAPDH) as an endogenous control (Rn99999916_s1, ABI, Carlsbad, CA). Analysis was first carried out using the 2^−ΔCT^ method.

### Western Blotting

All NPC samples were run on SDS-PAGE, and transferred to PVDF membranes which were incubated overnight in primary antibodies to SHH (1:200; Santacruz Biotechnology, Santa Cruz, CA), GDNF (1:500, Abcam, Cambridge, MA) or SDF1α (1:1000, Abcam, Cambridge, MA) and β-actin (1:2000; Santa Cruz Biotechnology, Santa Cruz, CA). Subsequently the membranes were exposed to the appropriate HRP conjugated secondary antibodies (Santa Cruz Biotechnology, Santa Cruz, CA). Blots were developed using ECL-Plus substrate (Amersham) and then scanned on Storm 860 Molecular Imager (GE Healthcare).

### RNA interference (please see Supplementary methods in S1 File for additional details)

For knockdown of SHH and GDNF, we produced five independent shRNAs against rat SHH or GDNF. The design of these shRNA sequences (mentioned in supplementary methods) followed the methods established in the Davidson lab [13, 14]. These shRNA’s were first co-transfected into 293T cells along with rat GDNF and rat SHH plasmids (generously provided by Dr Martha Bohn, Northwestern University, and Dr David Robbins, University of Miami respectively) using Lipofectamine 2000 (Invitrogen), to test the silencing efficiency of the shRNA constructs. After screening each of the shRNA constructs in the 293T cells for knockdown of SHH and GDNF, we selected 2 candidates, showing maximum silencing efficiency, from each group for further use in experiments. The selected shRNAs were then shuttled into feline immunodeficiency virus (FIV) lentiviral vectors with a GFP reporter at the Gene Transfer Vector Core at the University of Iowa [13]. eGFP expressing control vectors or non-targeted (scrambled) RNAi control vectors also were generated. Silencing efficiency of the shRNA vectors were tested at the mRNA (qPCR) and protein (western blot) levels.

### In vitro NPC viability and proliferation

NPCs were incubated with 0.4% trypan blue (Thermoscientific) and the number of live and dead cells enumerated to determine cell viability. To assess proliferation, the NPCs were incubated with 10μM brdU for 1 hour after which they were fixed with 4% paraformaldehyde. Subsequently the fixed cells were probed immunocytochemically using primary (brdU, Millipore-Chemicon, 1:200) and secondary (Alexafluor 488, Invitrogen, 1:500) antibodies.

### Animals and Experimental groups

The rats were housed in the vivarium at the University of Cincinnati, and treated according to the rules and regulations of NIH and Institutional Guidelines on the Care and Use of Animals. University of Cincinnati Institutional Animal Care and Use Committee specifically approved all experimental procedures. Experimental subjects were male Fischer 344 rats (250–275g, Harlan, Indianapolis, IN) which were maintained on a reverse 12 hour light-dark cycle with ad libitum food and water.

Experimental paradigm: NPCs were transplanted into the striatum and substantia nigra of the rats, one week before the 6-OHDA injections into the median forebrain bundle (MFB), and sacrificed at two weeks post-transplantation.

NPC transplanted animals were sacrificed using pentobarbital (60mg/kg), perfused with 4% paraformaldehyde (PFA), extracted brains post-fixed in 4% PFA solution, sunk through a 30% sucrose solution, and sectioned in the coronal plane (40 μm) on a freezing sliding microtome for morphological studies.

The number of animals in each experimental group were as follows:

HBSS + 6-OHDA (n = 7); NPCs (uninfected) + 6-OHDA (n = 8); NPCs (GFP control) + 6-OHDA (n = 8); NPCs (scrambled control) + 6-OHDA (n = 8); NPCs (shGDNF) + 6-OHDA (n = 11); NPC (shSHH) + 6-OHDA (n = 11); NPCs (shGDNF + shSHH) + 6-OHDA (n = 8).

### NPC transplants

Undifferentiated NPC’s (50,000 cells/μL of HBSS, 2 μL/site) were stereotaxically infused unilaterally into the left striatum (AP +0.2, ML +3.0, DV-5.5 from bregma) and SN (AP-5.3, ML +2.5, DV-7.0 from bregma), at a rate of 0.5ul/min, as described previously [5].

### 6-OHDA lesions

1 μl of 6-OHDA (4μg/μl) in a 0.2% solution of ascorbic acid in saline was infused unilaterally (ipsilateral to NPC grafted hemisphere) into the left median forebrain bundle (MFB, AP-4.0, ML +1.2, DV-7.5), using stereotaxic methods. The 6-OHDA infusion occurred at a rate of 0.5ul/min, and was conducted at 1 week after NPC transplantation [5].

### BrdU administration

Intraperitoneal bromodeoxyuridine (brdU) injections were used to label proliferating endogenous NPCs in the rats, at a dose of 50 mg/kg/12 hrs for 3 days before transplantation. Our previous studies have shown that administration of brdU before transplantation labels dividing NPCs in the germinal niches (SVZ and dentate gyrus of hippocampus) of the naïve brain, and allowed us to track the response of these endogenous precursors to exogenous NPC transplantation and 6-OHDA application (9).

### Behavioral analysis

To confirm success of nigrostriatal dopamine depletion, and/or degree of neuroprotection or recovery, rats were evaluated for spontaneous forepaw use with the cylinder task using established methods [5, 15]. The forelimb asymmetry score was calculated using the formula: (total ipsilateral limb use + 1/2 bilateral) / total limb use X 100.

### Immunohistochemistry (Antibody details in the supplementary section)

Standard protocols were used. Briefly, sections were blocked and incubated with the primary antibody overnight at room temperature (RT) or at 4°C for 48 hrs. Primary antibodies were detected in a 2-hr-incubation at RT with secondary antibodies (1:250) coupled to the fluorochromes Alexa Fluor 488, 555, 594, or 647 *(Invitrogen-probes*, *Carlsbad*, *CA)* for immunofluorescence. Alternatively, 3,3′ diaminobenzidine (DAB) or vector blue-staining with biotinylated secondaries and ABC peroxidase kit (Vector labs, Burlingame, CA) was performed. Sections probed for GFP (using the DAB technique) were counterstained with hematoxylin, for conducting counts of grafted NPCs.

### Stereology and Cell counts

#### Stereology

Stereological probes were applied using a BX52 Olympus microscope (Olympus America Inc.) equipped with Microbrightfield stereological software and a Microfire CCD camera (Optronics, Goleta, CA) using the optical fractionator method according to previously published methods [5]. Cells were counted under the 60X oil immersion objective. Tyrosine Hydroxylase (TH) cells were counted in sections 480 μm apart using a grid size of 170 X 100 μm and counting frame size of 50 X 50 μm. For brdU, counts were conducted through the dorsolateral SVZ in sections at 480 μm intervals between the genu of the corpus callosum and anterior commissure crossing. The grid size used was 100 X 100 μm and the counting frame was 75 X 75 μm. The Gundersen method for calculating the coefficient of error was used to estimate the accuracy of the optical fractionator results. Co-efficients obtained were generally less than 0.1.

#### Cell counts

For estimating the number of GFP^+^ cells expressing Tuj1 (neurons), S100ß (astrocytes), RIP (oligodendrocytes), and nestin (undifferentiated) within NPC grafts, confocal microscopy was used. Eight regions containing grafted cells (4 in graft center, and 4 in the graft periphery) were evaluated in 3 adjacent sections, under a 63X lens [5]. CD11b- and CD68-expressing microglia were also quantified in 3 adjacent sections containing grafted cells in each animal, with 4 regions in the graft periphery being evaluated under a 100X lens. Grafts in both the striatum and substantia nigra were evaluated in five animals per group. Data was expressed as mean ± SEM of percent of GFP^+^ cells expressing either Tuj1, S100ß, nestin or RIP, and the number of CD11b^+^ and CD68^+^ cells counted per section.

Grafted cell survival was estimated using the Microbrightfield’s Stereoinvestigator software using previously established protocols in 7–9 animals per group (5). Data was expressed as mean ± SEM of total GFP^+^ cells per animal.

### Microscopy

An OlympusBX60 light microscope with a NikonDXM1200 digital camera, or a Zeiss Axioplan 2 microscope with an Orca-ER cooled B&W CCD camera was used for fluorescence microscopy. A Zeiss LSM510 with Zeiss LSM software was used for confocal imaging/analysis in which Z sectioning (at 1–2 μm intervals) was conducted in order to verify the co-localization of markers.

### Statistical analyses

Sigmaplot 11 and Graphpad prism 5 software were used for statistical analyses. For comparisons between two or more groups, analysis of variance (ANOVA) followed by Dunnett’s post-hoc test for multiple comparisons with the control, or Tukey’s/Bonferroni’s test for multiple comparisons between treatment groups was conducted. Two-way repeated measures ANOVA (RM-ANOVA) was used to analyze the behavioral data. Differences were accepted as significant at *p* < 0.05. Specific statistical details as pertaining to each experiment are provided within the results and legend sections.

## Results

### An in vitro gene silencing approach to examine the role of GDNF and SHH in grafted NPC-induced nigrostriatal neuroprotection

**Fig 1:**
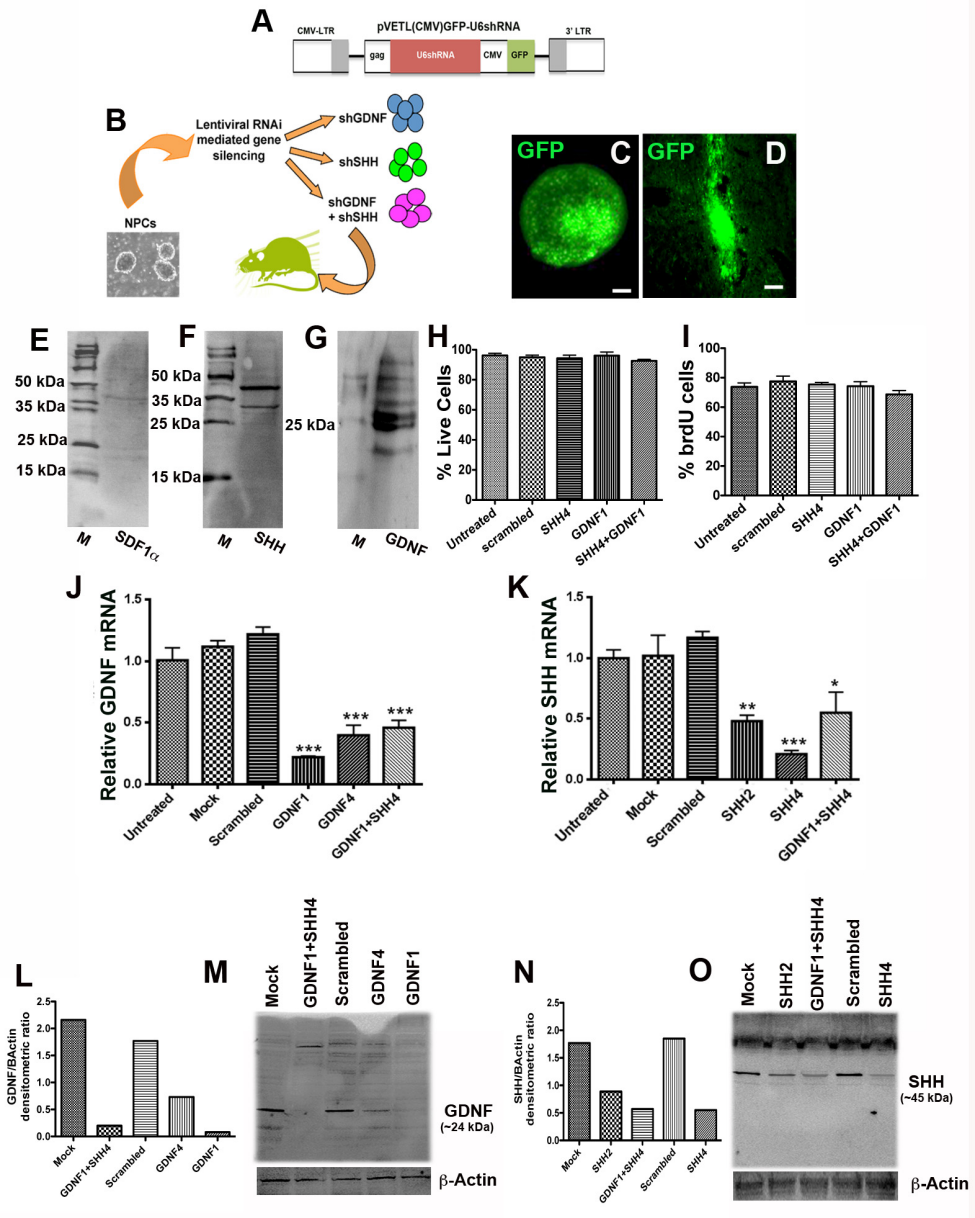
*In vitro* lentiviral silencing of GDNF and SHH in NPCs. Western blotting analyses of cultured NPCs indicated that the cells expressed only GDNF (~25kda) and SHH (~45kda), but no detectable SDF1α (~11kda), under basal conditions (E-G; GDNF and SHH were run on the same gel and membrane divided, whereas SDF1 was run on a separate gel). Therefore, an FIV based *in vitro* RNA interference approach was used to knock down the expression of GDNF, SHH or both in donor NPCs before transplantation (B). A schematic diagram of pVETL construct used for expression of shRNAs and the GFP reporter is depicted in (A). After testing, the two most efficient shRNA constructs targeting GDNF or SHH were cloned into a FIV eGFP vector and subsequently infected into the NPCs in culture (D, shows a representative image of *in vitro* NPCs infected with control eGFP vector). The NPCs did not show a significant change in survival and proliferative ability after the shRNA infections (H, I). When the silencing efficiency of the virally infected shRNA’s was examined through mRNA (qPCR; J, K) and protein (western blot; L-O) analyses, it was determined that in particular the GDNF1 and SHH4 constructs were able to most significantly (p<0.001) inhibit GDNF and SHH expression respectively, when compared to mock (eGFP vector) and scrambled (non-targeted shRNA) controls, and were focused upon in the rest of the study. Image E displays control FIV eGFP expressing NPCs *in vivo*, 2 weeks after transplantation. Values are expressed as mean ± SEM [*p<0.05, **p<0.01, ***p<0.001 compared to scrambled control, one-way ANOVA with Tukey’s post hoc test]. Scale bars: A, B, C (in C)– 25 μm; D, E—100 μm.

Our previous studies have determined that subventricular zone (SVZ) NPCs derived from the P0 postnatal rat brain, express three factors, namely SHH, GDNF, and stromal derived factor 1 alpha (SDF1α), and induce the protection of the host dopaminergic nigrostriatal system, when transplanted before the onset of 6-OHDA induced neurodegeneration [5] (A, B and C in S1 Fig). In order to determine whether and how these graft-expressed factors contributed to the observed nigrostriatal protection, we chose an *in vitro* lentiviral RNAi approach to silence GDNF, SHH or SDF1α in the NPCs before they were transplanted into recipient rats (Fig 1B). However, although all three of these molecules had been observed to be expressed by *in vivo* grafted NPCs under conditions of 6-OHDA induced neurodegeneration, when their basal expression was examined in *in vitro* cultured NPCs using western blotting it was noted that GDNF (~25 kDa) and SHH (~45 kDa) were expressed at clearly detectable levels, but SDF1α (~11 kDa) was not (Fig 1E–1G). Given this finding, which indicated that the NPC’s expression of SDF1α was dependent on injury relevant signals present in the 6-OHDA lesioned *in vivo* environment, we focused our efforts on only GDNF and SHH in the current study.

After testing multiple shRNA constructs targeting either of these two molecules (described in Supplementary methods in S1 File) four shRNAs, labeled shGDNF1, shGDNF4, shSHH2, shSHH4, showing maximum silencing efficacy, were transferred into FIV viral vectors, with a GFP reporter which enabled monitoring of in vitro silencing efficacy (Fig 1C) as well as in vivo tracking (Fig 1D) of the transduced cells. Fig 1A depicts the viral pVETLeGFP construct used for expression of the shRNAs. These shGDNF and shSHH vectors were subsequently, either individually or in combination, transfected into cultured NPCs and their silencing efficiency measured using qPCR and western blotting. Transfection of each of the four vectors resulted in significant knockdown of the target genes, however shGDNF1 (qPCR, p<0.001, F_*5*, *12*_ = 41.81, one way ANOVA) and shSHH4 (qPCR, p<0.001, F_*5*, *12*_ = 12.52, one way ANOVA) were most potent compared to mock (only GFP expressing) and scrambled (non-targeting) shRNA controls (Fig 1J–1O). The transfection rate of the GFP tagged lentiviral shRNA constructs into the NPCs was quite high with greater than 80% of cells expressing GFP after three days in vitro (Fig 1C, mean ± SEM of 26.4 ± 2.4 GFP cells among 32.0 ± 2.1 total cells per microscopic field analyzed). Also co-infection of the most potent GDNF and SHH shRNA viral constructs resulted in a significant knockdown of both these molecules in the NPCs (Fig 1J–1O). In this case, although the mRNA levels of GDNF and SHH were higher in the co-infected samples than samples in which only SHH or GDNF had been transduced, these differences were not statistically significant, and the western analysis showed a robust knockdown of both GDNF and SHH upon con-infection. We also evaluated the viability and proliferation rate of the cultured NPCs following viral shRNA infection. As shown in Fig 1H and 1I, trypan blue and brdU pulse labeling assays showed that the viability and proliferative capacity of the silenced NPCs was not significantly altered after the shRNA infections. Furthermore, as depicted in the supplementary S1D–S1W Fig, following transplantation, grafted NPCs expressing these specific shRNA constructs exhibited significantly reduced expression of GDNF and SHH *in vivo*.

### Silencing of SHH in grafted NPCs interferes with their ability to induce nigrostriatal neuroprotection and endogenous neurogenesis

**Fig 2:**
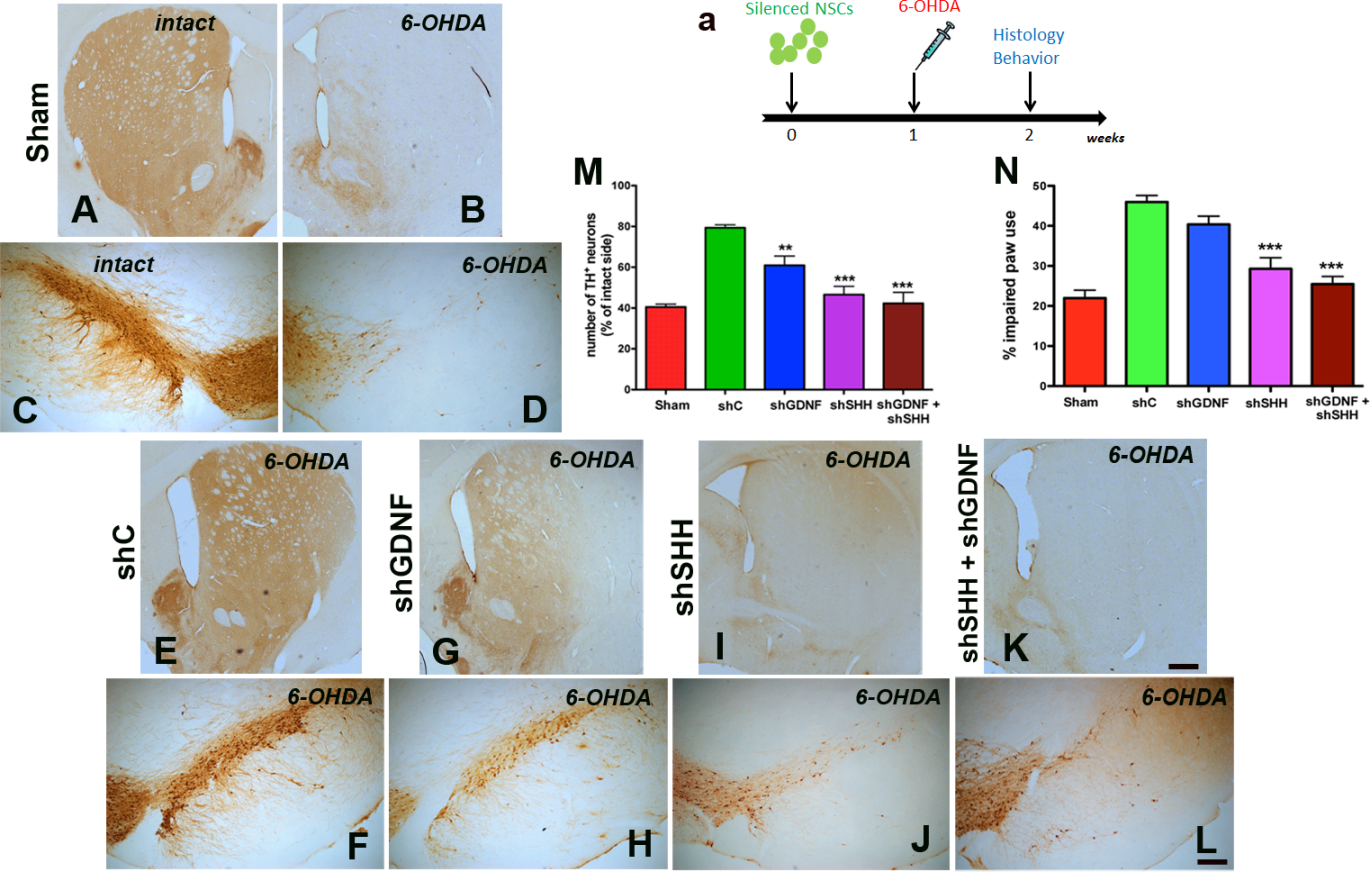
SHH silencing inhibits the neuroprotective effects of donor NPCs. NPCs carrying shRNA’s directed at GDNF, SHH or both were implanted into the striatum and SN of Fisher 344 rats 1 week before the administration of 6-OHDA (a). Behavioral assessment using spontaneous paw placement in the cylinder task (N), at two weeks after NPC implantation indicated that animals which had received NPCs with non targeted control shRNA (shC) displayed preservation of the function of the affected forepaw (contralateral to 6-OHDA lesion) compared to animals receiving no cells (just medium). However, rats receiving SHH silenced grafts (shSHH or both shSHH+shGDNF) showed a significant reduction in the ability to use the affected forepaw, similar to sham rats. GDNF silencing did not have any effect on the therapeutic benefit of NPC grafts in the forepaw task (N). Immunohistological staining showed that the preservation of TH immunoreactivity in the striatum (E) and SN (F) observed in the control NPC transplanted group was visibly reduced in the animals with shSHH and shSHH+shGDNF silenced grafts (I, J, K, L). Animals that had received shGDNF NPC grafts showed partial preservation of TH immunoreactivity in the striatum (G) and SN (H) compared to shSHH (I, J) and shSHH+shGDNF (K, L) grafted animals. Stereological estimates of TH^+^ cells in the SN also showed a similar pattern with the shSHH and shGDNF+shSHH showing greatest potential to reduce the neuroprotective effect of the control NPCs. Values are expressed as mean ± SEM [**p<0.01, ***p<0.001 compared to sham controls, two way RM-ANOVA with Tukey’s post-hoc test for behavioral data in N, one way ANOVA with Bonferroni’s post-hoc test for stereological estimates in M]. Scale bar: 500 μm

NPCs infected with the most efficient shRNA constructs targeting either GDNF (shGDNF1), SHH (shSHH4), both (shGDNF1 + shSHH4), and controls [only GFP and scrambled (referred to as shcontrol; shC)] were transplanted into the striatum and SN of host rats, in which 6-OHDA was administered one week after NPC implantation (Fig 2A). When the animals were histologically examined 2 weeks after NPC transplantation (Fig 2A), it was observed that implantation of NPCs harboring the control shRNA induced nigrostriatal neuroprotection (Fig 2E and 2F) in comparison to sham injected animals (Fig 2A–2D). However, NPCs in which GDNF or SHH had been silenced showed a significantly reduced ability to induce neuroprotection (Fig 2G–2J). In particular, the knockdown of SHH alone or in combination with GDNF caused a profound reduction in NPC-mediated neuroprotection noted both the lateral tier of substantia nigra, which houses the PD relevant A9 dopamine neurons, and the striatum (Fig 2I–2L), when compared to the control (Fig 2E and 2F) and silencing GDNF alone (Fig 2G and 2H). Stereological quantification of the number of TH^+^ neurons in the SN supported the qualitative histological observations (Fig 2M, p<0.0001, *F*_4, 39_ = 14.85, one way ANOVA), and confirmed that the silencing of SHH alone (p<0.001), or in combination with GDNF (p<0.001), showed maximum efficiency in diminishing the neuroprotective ability of the NPCs compared to control cells (raw stereological counts in Table A in S1 File). Analysis of lesion-induced asymmetry of spontaneous forepaw use in the cylinder task, supported the histological data indicating that the silencing of SHH, or SHH and GDNF together, were most effective in decreasing the neuroprotective potential of the NPCs at a behavioral level (Fig 2N, p<0.001, *F*_*4*, *40*_ = 18.64, two way RM-ANOVA). We also mention here that no significant differences existed between the shGDNF+shSHH and shSHH groups, both in terms of TH cell counts and the behavioral data.

**Fig 3:**
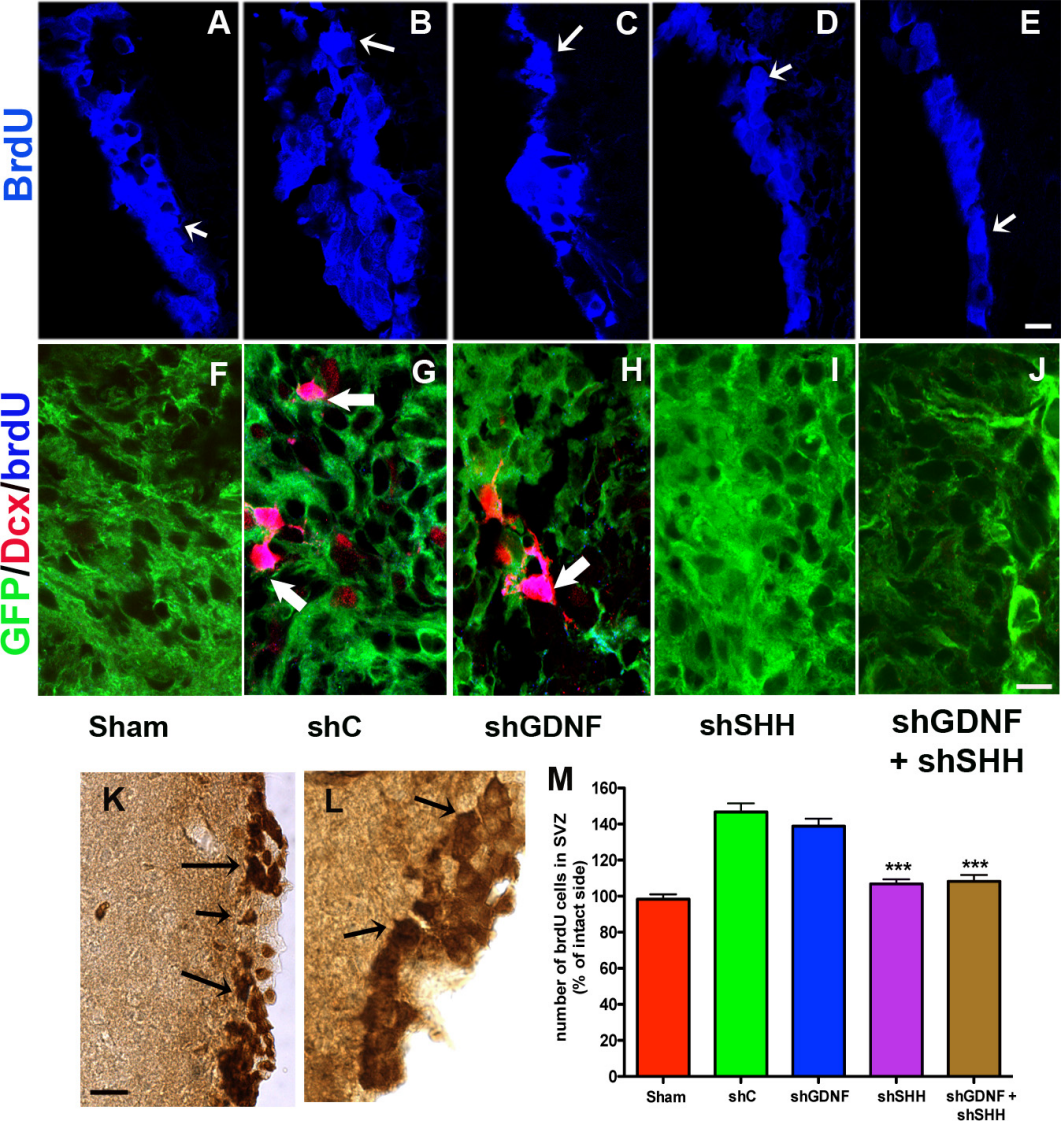
SHH silencing reduces grafted NPC-mediated endogenous neurogenesis. Effects of SHH and GDNF silencing on grafted NPC-induced endogenous neurogenesis and neuroblast migration were examined. As shown in A-E, in comparison to animals with control NPC implants, those transplanted with SHH and GDNF+SHH silenced NPCs showed reduced brdU labeling within the host forebrain SVZ niche. In support of these data, cell counts indicated lower numbers of brdU^+^ cells in the ipsilateral SVZ of rats that had received SHH or GDNF+SHH silenced NPC transplants (arrows, examples of DAB labeled cells counted in K, L, stereological counts in M). Further, migrating brdU^+^/dcx^+^ neuroblasts, which were observed in the striatal parenchyma, particularly in regions neighboring grafts, in control NPC receiving animals (arrows, G), were missing in those with SHH or GDNF+SHH silenced cells (I, J). With regard to animals harboring GDNF silenced cells, a significant reduction in brdU^+^ cells (M) or an absence of brdU^+^/dcx^+^ cells in the lesioned and grafted striatum (H) was not observed. Values are expressed as mean ± SEM [***p<0.001 compared to shC, one way ANOVA with Bonferroni’s post-hoc test]. Scale bars: A-E—50 μm, F-J—10 μm, K, L—50 μm.

In addition to neuroprotection, we assessed cellular proliferation in the host SVZ through the stereological quantification of brdU labeled cells (arrows in Fig 3K and 3L), given the observation that the NPC grafting induced endogenous neurogenesis in our previous studies (Madhavan et al, 2009). Here, it was noted that the transplantation of control cells resulted in a significant escalation in the proliferation of endogenous precursor cells in the SVZ of the lesioned hemisphere compared to sham treatment (Fig 3A, 3B and 3M, p<0.001, *F*_*4*, *43*_ = 31.57, one way ANOVA). However, the number of brdU^+^ cells was significantly lower in the SVZ of animals transplanted with NPCs in which SHH, or SHH and GDNF were down-regulated (p<0.001, Fig 3D, 3E and 3M) (raw stereological counts in Table A in S1 File). BrdU cell numbers in the shSHH group were determined to be not significantly different from those in the combined shGDNF+shSHH group. On the other hand, GDNF-only silencing did not affect host proliferation and neurogenesis, and animals receiving the GDNF-only silenced cells exhibited SVZ brdU^+^ cell counts similar to animals in the control group (p<0.001, Fig 3C and 3M). We also observed endogenous brdU labeled cells expressing doublecortin (dcx, a marker of migrating neuroblasts) in striatum, particularly adjacent to the grafts of control NPCs (Fig 3G). Such brdU^+^/dcx^+^ cells were absent in animals that had received SHH, or SHH+GDNF silenced cells (Fig 3I and 3J), although the double-labeled cells were still observed in rats receiving grafts lacking GDNF (Fig 3H).

### SHH silencing alters the phenotype of grafted NPCs

**Fig 4:**
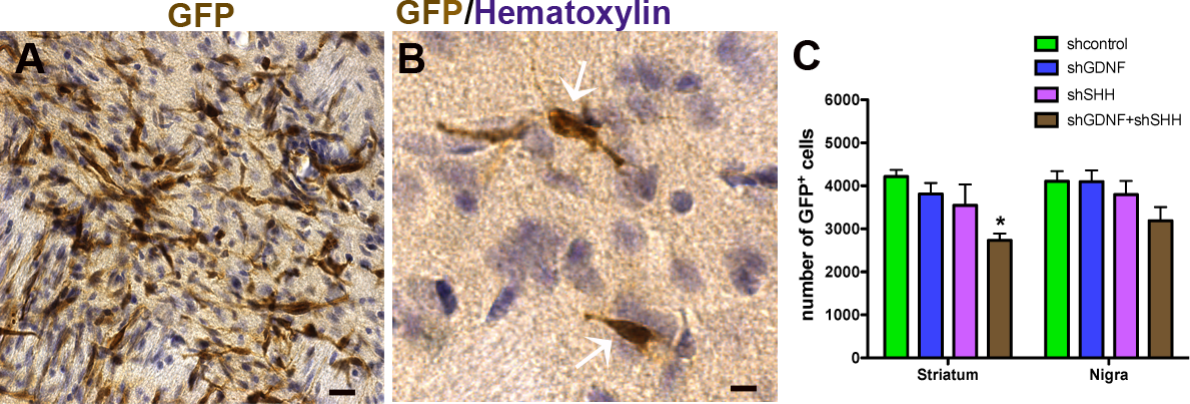
Effect of SHH and GDNF silencing on the number of grafted NPCs. We assessed the number of GFP expressing donor cells (A) in both the striatum and nigra of rats receiving control and silenced NPCs. Estimates of grafted GFP^+^ cells in hematoxylin counterstained tissue sections (B) showed no significant differences in the striatum or SN of SHH or GDNF silenced animals when compared to controls. However the animals receiving GDNF+SHH silenced grafts showed reduced numbers of grafted cells in the striatum in comparison to animals with control cells (C). Values are expressed as mean ± SEM [*p<0.05, **p<0.01, ***p<0.001 compared to shC, two way ANOVA with Bonferroni’s post test].Scale bars: A—25 μm; B—10 μm.

To further characterize the effects of SHH and GDNF knockdown, the number of grafted NPCs in both the striatum and SN was assessed at two weeks after NPC transplantation. Enumeration of GFP expressing grafted cells (Fig 4A and 4B) indicated no significant differences in grafted NPC numbers in the striatum when SHH (3,548 ± 486.8) or GDNF (3,810 ± 254.1) had been silenced when compared to control animals (4,217. ± 236.6) (Fig 4C, p>0.05, *F*_*3*, *58*_ = 5.92, two way ANOVA). In comparison, GDNF+SHH silenced animals showed lower numbers of GFP^+^ grafted NPCs (2,734 ± 159.1). In the SN, the number of grafted NPCs among the different experimental groups did not show significant differences (shC: 4,104 ± 236.6; GDNF: 4,099 ± 261.9; SHH: 3,795 ± 379.9). Also, GFP cell counts conducted in a cohort of animals at a later time point of 5 weeks (n = 4), indicated that the number of grafted cells in the SHH and GDNF silenced groups were not significantly different at this stage from the two week time point (SHH: Striatum—3,605 ± 414.4, Nigra—3,986 ± 235.3; GDNF: Striatum—3,991 ± 120.62, Nigra—3,982 ± 117.5). Thus, we found no evidence of ongoing degeneration of grafted cells at 5 weeks post-implantation. In terms of migration, the grafted cells remained largely confined to the site of implantation, and there were no significant differences observed in the location of grafted cells between the control and experimental groups.

**Fig 5:**
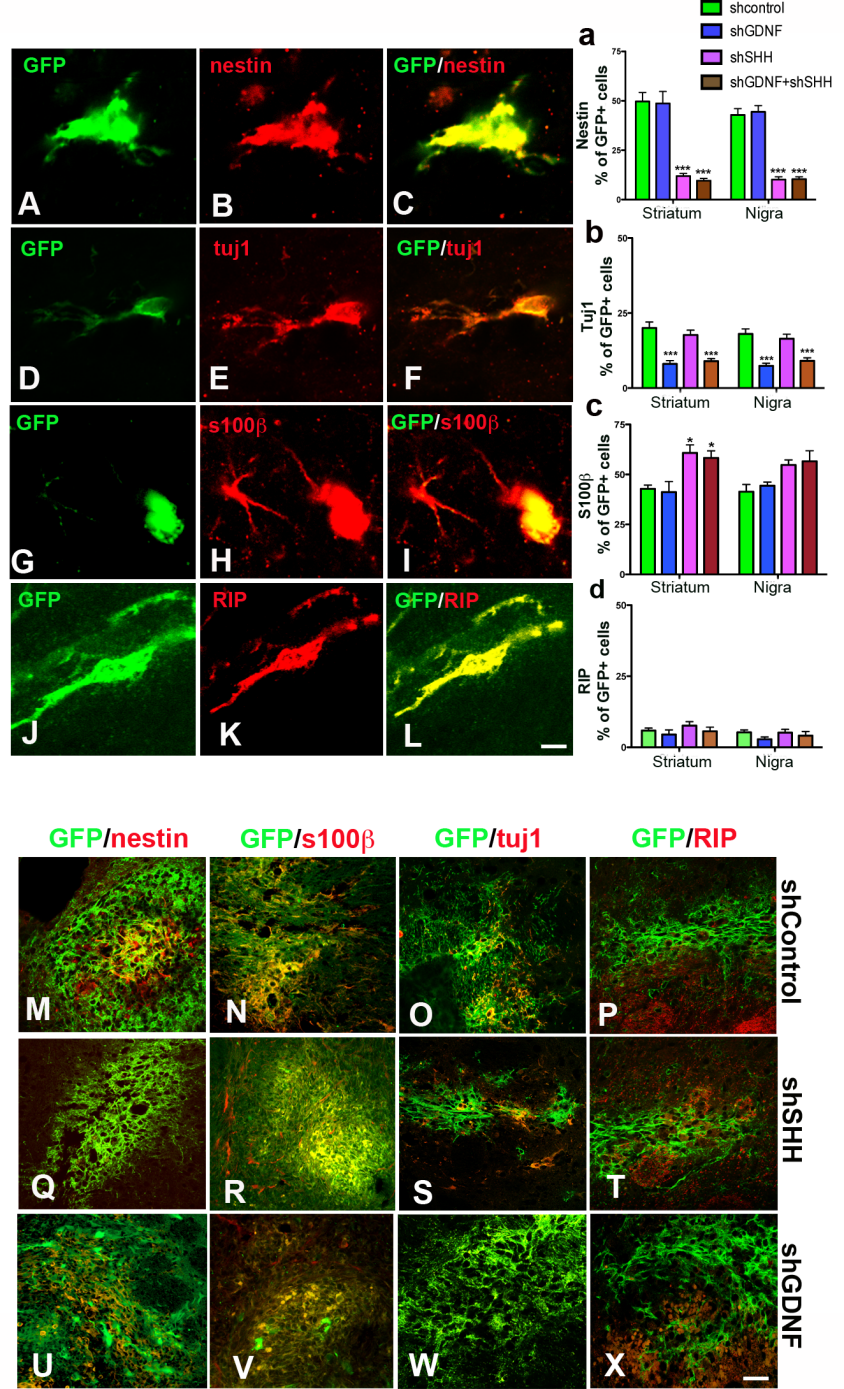
Inhibition of SHH influences the phenotypic fate of grafted NPCs. We analyzed the co-expression of antigens specific to nestin (undifferentiated NPCs, A-C), immature neurons (tuj1, D-F), astrocytes [(s100β (G-I) and oligodendroglia [RIP (J-L)] in the GFP NPCs. Counts of grafted GFP cells indicated that while the number of nestin^+^ cells was significantly lower, compared to control NPCs in donor cells silenced for SHH or GDNF and SHH, shGDNF grafts did not show such an effect (a). This was true for grafts both in the striatum as well as SN. Representative images of nestin stained grafts are shown in M, Q, U. The GDNF silenced grafts on the other hand showed a significant (p<0.001) reduction in the number of cells expressing the neuron marker tuj1 (b). Such a decline in tuj1 expression was not observed in the SHH silenced NPCs. Representative images of tuj1 stained grafts are shown in O, S, W. Further, the number of S100β^+^ cells was greater in striatal grafts with SHH knock-down, with no such influence observed in any of the other experimental groups (c). Representative images of S100β^+^ cells in grafts are shown in N, R, V. Finally, with respect to oligodendrocyte differentiation, no significant differences were observed between control and silenced NPCs (d). Representative images of RIP staining in grafts are shown in P, T, X. Values are expressed as mean ± SEM [*p<0.05, **p<0.01, ***p<0.001 compared to shC, one way ANOVA with Bonferroni’s post test]. Scale bars: A-L—10 μm; M-X—30 μm.

**Fig 6:**
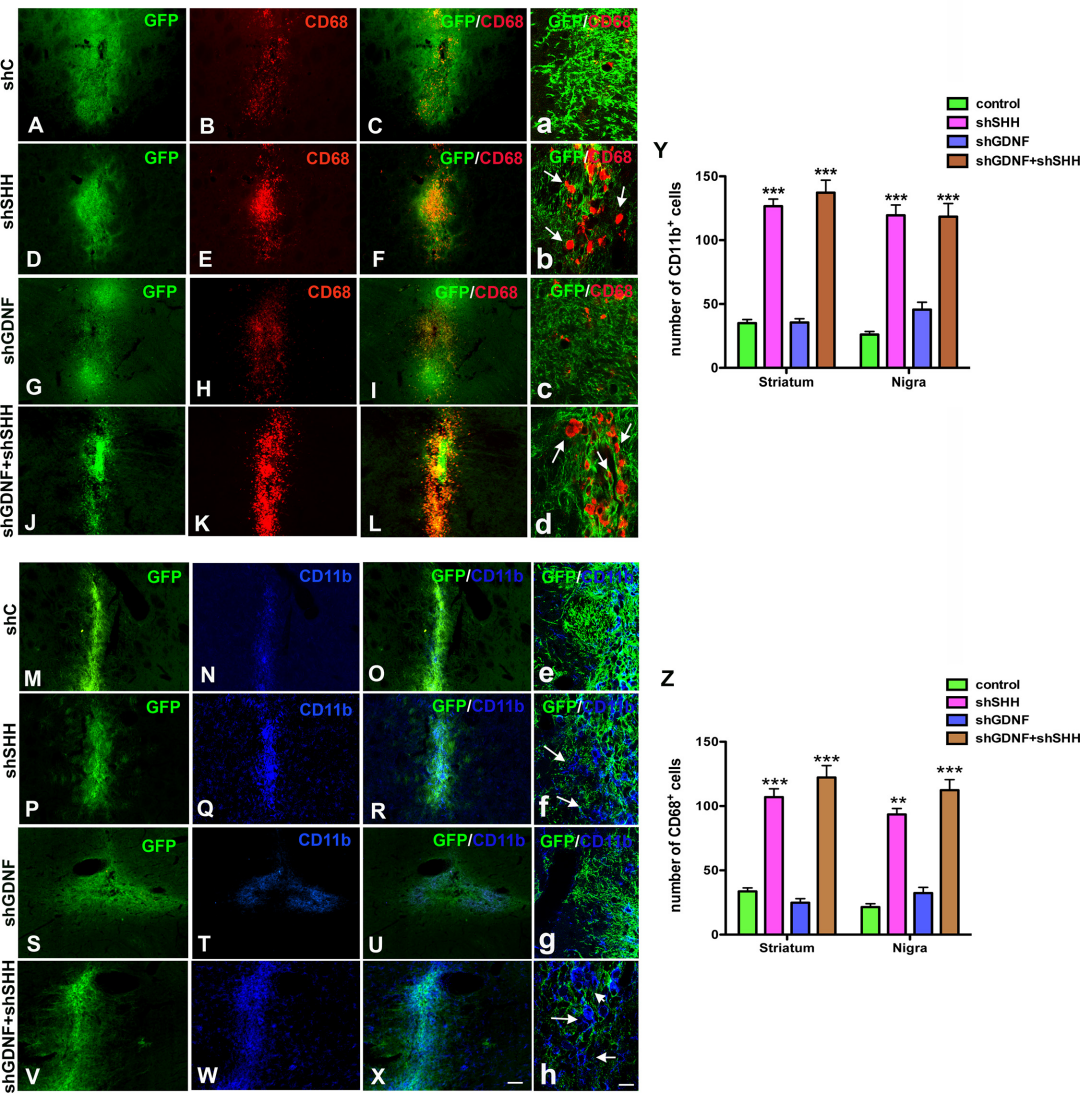
SHH knockdown increases microglial activity in the host brain. Host microglia expressing cd11b and cd68 antigens (expressed by activated microglia) were analyzed two weeks after NPC implantation. A greater number of both cd68^+^ and cd11b^+^ cells were noted in animals that had received the SHH or GDNF+SHH silenced NPCs (D-F, J-L, P-R, V-X high mag images in b, d, f, g) in comparison to rats with control (A-C, M-O; high mag images in a and e) or GDNF silenced (G-I, S-U; high mag images in c and g) grafts. The number of cd11b^+^ and cd68^+^ cells present in regions adjacent to the transplanted NPCs were quantified using confocal microscopy, and it was confirmed that the decrement of SHH in grafted NPCs had in fact resulted in a significant (p<0.001) activation of microglia in the host neural environment (Y, Z). Values are expressed as mean ± SEM [***p<0.001 compared to control, one way ANOVA with Bonferroni’s post test]. Scale bar: A-S—50 μm, a-f—15 μm.

The phenotype of grafted NPCs was examined via immunohistochemical tagging of the GFP cells with NPC (nestin, Fig 5A–5C), as well as neuronal (tuj1, Fig 5D–5F and 5B) and glial (S100β, RIP, Fig 5G–5L, 5C and 5D) antigens. Interestingly, compared to control and GDNF silenced cells, grafted NPCs in which SHH or SHH and GDNF had been silenced showed a significant reduction in the expression of nestin, which is a marker of undifferentiated NPCs. As shown, reduced nestin staining was detected both in intra-striatal grafts and grafts in the SN (Fig 5M, 5Q and 5U; graph in Fig 6A—p<0.001, *F*_*7*, *112*_ = 35.38, one way ANOVA) [Number of nestin^+^ cells among GFP^+^ cells per section analyzed (mean ± SEM): shC Striatum—59.8 ± 4.6 in 125.1 ± 9.2; shSHH Striatum—14.2 ± 1.4 in 119.7 ± 9.3; shCNigra—52.2 ± 4.0 in 124.5 ± 8.8; shSHH Nigra—10.0 ± 1.1 in 109.2 ± 10.5]. No statistically significant differences were detected in nestin cell numbers, between the shSHH and shGDNF+shSHH groups, in the striatum or SN. In terms of neuronal differentiation, tuj1^+^ cell numbers were significantly reduced in grafts which had been silenced for GDNF (Fig 5O, 5S and 5W; graph in Fig 5B—p<0.001, *F*_*7*, *112*_ = 14.58, one way ANOVA), but the silencing of SHH did not produce significant alterations in numbers of neuron marker-positive grafted cells in the striatum or in the SN [Number of tuj1^+^ cells among GFP^+^ cells per section analyzed (mean ± SEM): shC Striatum—32.5 ± 2.7 in 168.5 ± 10.2; shSHH striatum—9.3 ± 1.0 in 128.1 ± 9.4; shC Nigra—29.6 ± 3.0 in 164.5 ± 7.9; shSHH Nigra—11.8 ± 1.0 in 168.9 ± 11.8]. Here, the shGDNF and shGDNF+shSHH groups showed similar tuj1 cell numbers, however significant differences existed between shSHH and shGDNF+shSHH groups. On examination of the glial fate of the grafted NPCs, it was observed that SHH knockdown resulted in a significant expansion of the S100β^+^, presumed astrocyte, cell population within the striatal grafts but not in the SN (Fig 5N, 5R and 5V; graph in Fig 5C—p<0.05, *F*_*7*, *112*_ = 5.044, one way ANOVA) [Number of s100β^+^ cells among GFP^+^ cells per section analyzed (mean ± SEM): shC Striatum—35.0 ± 2.9 in 83.0 ± 7.2; shSHH striatum—47.6 ± 3.8 in 83.5 ± 8.8]. These altered astrocytic cell numbers in NPC grafts did not significantly differ between the SHH and GDNF+SHH silenced animals, in either the striatum or SN. A similar number of RIP expressing cells were observed in grafts in all the experimental groups, both in the striatum and the SN, indicating that oligodendrocyte differentiation potential of the NPCs was minimal and had not been altered by the silencing of SHH or GDNF (Fig 5P, 5T, 5X and 5D).

### SHH silencing in grafted NPCs modulates the host glial reaction

We examined the endogenous immune reaction to grafting by analyzing the presence of both CD11b and CD68 (activated) expressing microglia using immunohistochemical methods. Analyses indicate that both CD11b^+^ and CD68^+^ cells were present in greater numbers within and adjacent to grafts in animals transplanted with NPCs in which SHH, or both SHH and GDNF, had been silenced (Fig 6D–6F, 6J–6L and 6P–6R, V-X; high magnification confocal images in Fig 6B, 6D, 6F and 6H). No significant statistical differences were determined in microglial cell numbers between the shSHH and shGDNF+shSHH groups of animals. Control NPC grafted animals on the other hand had lower numbers of microglial cells in regions neighboring the implanted NPCs (Fig 6A–6C, Fig 6M–6O; high magnification confocal images in Fig 6A and 6E). Moreover, transplantation of the GDNF silenced NPCs did not result in an increase in microglia when compared to controls (Fig 6G–6I, 6S–6U; high magnification confocal images in 6C and 6G), and significantly lower numbers of microglia were determined in this group of animals compared to animals in the shSHH and shGDNF+shSHH groups (p<0.001, one way ANOVA). Quantification of the number of microglial cells adjacent to grafted NPC regions supported the qualitative impressions indicating that specifically SHH played a role in inhibiting the response of endogenous CD11b^+^ (p<0.001, *F*_*7*, *112*_ = 81.79, one way ANOVA) and CD68^+^ (p<0.001, *F*_*5*, *84*_ = 80.74, one way ANOVA) cells after transplantation (Fig 6Y and 6Z).

## Discussion

Diffusible growth and plasticity promoting factors, expressed by grafted NPCs, are implicated as important molecules governing a range of therapeutic effects known to occur after NPC transplantation. More specifically, studies indicate that the expression of such factors in transplanted NPCs is associated with neuroprotective and restorative effects in many models including those of PD, stroke, and immune dysfunction [4, 16–18]. Yet, the precise relationship between the expression of defined factors in donor NPCs and therapeutic effects observed *in vivo* is not well understood. In particular, it is not clear whether, how, and to what degree, the NPC-expressed factors influence subsequent therapeutic sequelae. In this context, our study is novel in that it uses focused RNAi methods to examine the participation of specific graft-expressed molecules, and helps explain how a donor factor like SHH significantly impacts both graft and host function to mediate therapeutic effects in this model of nigrostriatal neurodegeneration.

A loss in SHH expression in donor NPCs significantly compromised the neuroprotective ability of grafted NPCs in our study. In addition, the SHH deficiency also diminished endogenous neural precursor cell proliferation and neurogenesis in the host brain—a phenomenon which was preserved after the grafting of non-silenced control NPCs which expressed SHH. In support of these findings, it is known that SHH is crucial for the survival and differentiation of developing as well as adult midbrain dopamine neurons [19–23]. In addition, SHH is also recognized to be fundamentally involved in the maintenance and regulation of adult neurogenic niches [24–27]. The current study provides evidence that these important properties of SHH also function in a local “ectopic niche” associated with grafted NPCs, participating in SN DA neuron protection and directed proliferation and neurogenesis in the SVZ, adding another facet to SHH’s plastic capabilities. Moreover, our study also implicates GDNF as a minor contributor to the observed nigrostriatal neuroprotection, although it does not support a role for the factor in stimulating SVZ neurogenesis or endogenous NPC migration. This is interesting given that GDNF is recognized to be an important factor capable of influencing midbrain DA neuron survival and differentiation, and neurogenesis in the adult brain [28–31]. It is possible that the quantity of GDNF expressed by the graft was not sufficient to induce a strong effect in our model. The loss of GDNF did have an influence on the phenotypic fate of the grafted NPCs by reducing neuronal differentiation, nevertheless it did not have the profound influence of SHH, which appeared to strategically act on both grafted and host cell populations to affect their phenotype and function, and subsequently shape therapeutic consequences.

SHH also affected the phenotypic fate of grafted NPCs in our study. It was observed that grafted cells in which SHH had been silenced showed no changes in survival after transplantation, but exhibited altered differentiation. In particular, silencing of SHH caused a striking decline in the number of nestin^+^ (undifferentiated) cells, but an increase in S100β^+^ glia, in the donor NPCs. Indeed both nestin and SHH are recognized as being key molecules regulating NPC survival, self-renewal/proliferation, and differentiation [25, 32]. The present study supports this concept, and indicates that the nestin-SHH expressing NPC population may be the principal cell type contributing towards neuroprotective and neurogenic effects in the PD model. The data also suggest that the loss of SHH induced greater differentiation of the grafts, in particular towards a glial fate, causing a reduction in the important undifferentiated nestin^+^ cells. Further, in terms of survival, grafts with NPCs silenced for both GDNF and SHH showed reduced cell numbers in the striatum, but were however equally potent to SHH silenced cells in reducing the efficacy of neuroprotection. This could possibly be attributed to the better survival of these grafts in the SN, which could have compensated to some extent, and perhaps a combinatorial effect of the loss of both GDNF and SHH.

In addition to implicating a significant role for SHH in the observed neuroprotection and neurogenesis, the current study also implicates a role for this factor in modulating the activity of host cd11b^+^ and cd68^+^ microglia [33]. In this context, reciprocal interactions between NPCs and microglia have been described previously [34, 35]. In particular, it has been shown that transplanted NPCs can reduce the proportion of M1 macrophages, leading to an alteration of the local inflammatory environment from a ‘hostile’ to an ‘instructive’ state [36]. Moreover it is known that the M1/M2 macrophage ratio critically modulates nervous system injury and repair [37, 38]. A similar mechanism could be at play in our study, wherein the loss of bonafide nestin-SHH NPCs inhibited such immune modulatory NPC effects to result in an environment harboring a pro-inflammatory microglial phenotype and leading to reduced graft function. In support of this idea, recent reports demonstrate that SHH can control the immune response by decreasing the expression of pro-inflammatory mediators such as TNF*α* and IL1*β*, and reducing the adhesion/migration of leukocytes [39, 40].

In conclusion, our report clearly identifies contributions of a specific graft-expressed factor, SHH, in the protection of host midbrain TH^+^ neurons after NPC transplantation. The protective effects of SHH were most pronounced in the environment of the dopamine terminals in the striatum, maintaining grafted cells in an undifferentiated state (nestin^+^), stimulating proliferation and migration of host SVZ NPCs, and inhibiting local activation of microglia. It remains to be determined whether the benefits of local augmentation of SHH signaling is specific for dopamine neurodegeneration, or can be generalized to other forms of brain injury and disease, by recapitulating aspects of developmental plasticity, stimulating and attracting endogenous NPCs, and reducing negative consequences of inflammation. Understanding the molecular dynamics of such SHH mediated graft-host signaling may have significant implications towards developing therapeutic approaches for Parkinson’s disease or more broadly applied in strategies supporting brain protection and repair.

## Supporting Information

S1 Fig Determination of SHH and GDNF silencing in donor NSCs after grafting. As observed in our previous studies, GDNF, SHH, and SDF1α were expressed by control (unsilenced) NPCs *in vivo*, at 2 weeks post-transplantation into 6-OHDA lesioned rats (A, B, C). In grafted NPCs which had been silenced for either GDNF, SHH, or a combination of GDNF & SHH, before implantation, immunohistochemical analysis of transplanted NPCs at 2 weeks indicated that the GFP positive donor NSCs transduced with shGDNF showed very low expression of GDNF (D-F). High magnification confocal images confirmed this (G-I). Similarly grafted NPCs infected with shSHH showed significantly diminished expression of SHH (J-L, high mag confocal images in M-O), whereas NPCs silenced for both GDNF and SHH showed reduced expression of both these factors (P-S, high mag confocal images in T-W).
(TIF)

S1 File Includes (1) Table A (Stereological TH+ and BrdU+ cell counts corresponding to the graphs in Figs 3M and 4M respectively) and (2) Supplementary methods. (DOCX)
