# Supplementary figures and images for: Sonic Hedgehog Controls the Phenotypic Fate and Therapeutic Efficacy of Grafted Neural Precursor Cells in a Model of Nigrostriatal Neurodegeneration
Source: PLoS One. 2015 Sep 4;10(9):e0137136. doi: 10.1371/journal.pone.0137136 (PMC4560385; doi:10.1371/journal.pone.0137136)

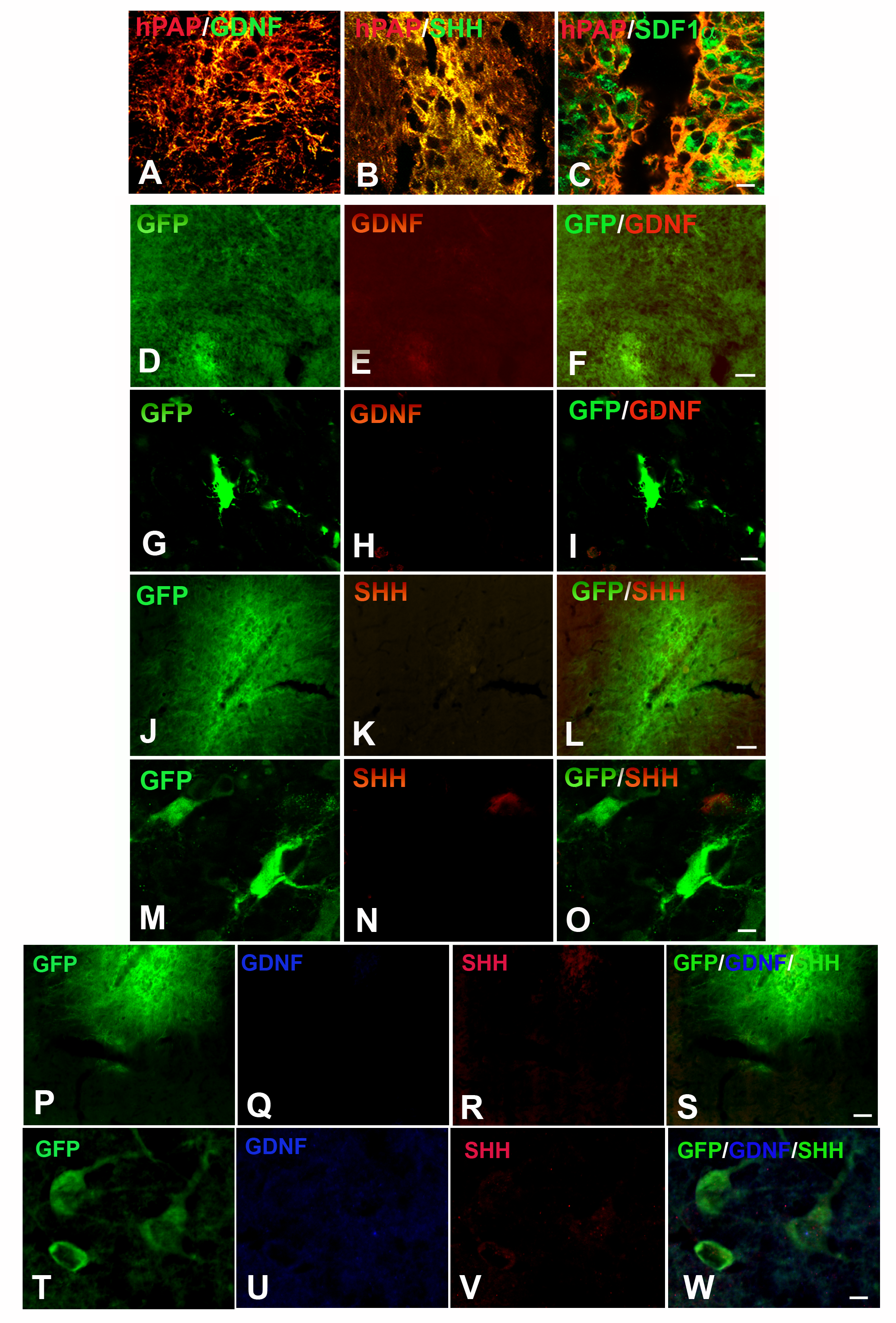

Supplement: S1 Fig — As observed in our previous studies, GDNF, SHH, and SDF1α were expressed by control (unsilenced) NPCs in vivo, at 2 weeks post-transplantation into 6-OHDA lesioned rats (A, B, C). In grafted NPCs which had been silenced for either GDNF, SHH, or a combination of GDNF & SHH, before implantation, immunohistochemical analysis of transplanted NPCs at 2 weeks indicated that the GFP positive donor NSCs transduced with shGDNF showed very low expression of GDNF (D-F). High magnification confocal images confirmed this (G-I). Similarly grafted NPCs infected with shSHH showed significantly diminished expression of SHH (J-L, high mag confocal images in M-O), whereas NPCs silenced for both GDNF and SHH showed reduced expression of both these factors (P-S, high mag confocal images in T-W). (TIF) [file pone.0137136.s001.tif]
