# Supplementary material for: Sonic Hedgehog Controls the Phenotypic Fate and Therapeutic Efficacy of Grafted Neural Precursor Cells in a Model of Nigrostriatal Neurodegeneration
Source: PLoS One. 2015 Sep 4;10(9):e0137136. doi: 10.1371/journal.pone.0137136 (PMC4560385; doi:10.1371/journal.pone.0137136)
Supplement: S1 File — (DOCX) [file pone.0137136.s002.docx]

**Table A:** Stereological TH+ and BrdU+ cell counts corresponding to the graphs in figures 3M and 4M respectively. I=Intact hemisphere; L=Lesioned hemisphere

| **Relates to Figures 2 and 3** | **Sham** | **shC** | **shSHH** | **shGDNF** | **shSHH + shGDNF** |
| --- | --- | --- | --- | --- | --- |
| **TH+ cells (Fig 3M)** | 14,890.14 ± 954.43 (I)  6098.71 ± 581.41  (L) | 13,869.12 ± 1,849.05 (I)  11,717.12 ± 2,004.51 (L) | 13,466 ± 1,342.9  (I)  6,147.5 ± 664.1  (L) | 13,398.2 ± 1,024.7 (I)  8,098.2 ± 842.1  (L) | 16.177.28 ± 1,991.75 (I)  6809.71 ± 1,076.05  (L) |
| **BrdU+ cells (Fig 4M)** | 20,155.42 ± 1,176.86 (I)  19,836.57 ± 1,306.47 (L) | 17,077.87 ± 1140.64 (I)  25,090.5 ± 1,849.94 (L) | 18,524.3 ± 1,782.6  (I)  19,727.9 ± 1,956.7  L) | 15,817.5 ± 1,238.36  (I)  23,049.4 ± 2,045.23  L) | 17,29.57 ± 1,925.40  (I)  18,758.28 ± 2,403.24 (L) |

**Supplementary methods:**

**shRNA generation:**

To begin with, five independent shRNAs against rat SHH or GDNF (sequences mentioned below) were generated utilizing the principles and methods established Dr Beverly Davidson’s lab at the University of Iowa (13, 14).

| GDNF  gccaccaucaaaagacugaacuguaaagccacagaugggUUCAGUCUUUUGAUGGUGGCuu  ggaggaacugaucuuucgaucuguaaagccacagaugggAUCGAAAGAUCAGUUCCUCCuu  gcggccgagacaauguacgacuguaaagccacagaugggUCGUACAUUGUCUCGGCCGCuu  ggcuaacaagugacaagguacuguaaagccacagaugggUACCUUGUCACUUGUUAGCCuu  gcauuccgcuaaacggugugcuguaaagccacagaugggCACACCGUUUAGCGGAAUGCuu |
| --- |
| SHH  gcugaccccuuuagccuauaccguaaagccacagaugggUGUAGGCUAAAGGGGUCAGCuu  gggaagaucucacaagaaaUccguaaagccacagaugggGUUUCUUGUGAGAUCUUCCCuu  ggaugaggaaaacacuggagccguaaagccacagaugggUUCCAGUGUUUUCCUCAUCCuu  ggucuucuacgugaucgagaccguaaagccacagaugggUUUCGAUCACGUAGAAGACCuu  ggugccaagaaggucuuUuaccguaaagccacagaugggUAGAAGACCUUCUUGGCACCuu |

Next, U6 promoter based shRNA constructs were generated using the PCR-based approach described previously Harper et al., 2005 (13). More specifically, the designed shRNA oligos were synthesized (Sigma-Aldrich) after which a one step PCR method was used to generate the U6shRNA expression cassette. Following PCR amplification the shRNA product was cloned into a pCR vector using the zero blunt TOPO PCR cloning kit (Invitrogen), transformed in bacteria, and clones selected and analyzed by restriction digestion and DNA sequencing. These constructs were screened and tested in 293T cells using the following methods.

293T transfections: 293T cells were grown in 24-well plates coated with poly-l-ornithine (0.1 mg/ml; Sigma, St Louis, MO) and transfected in quadruplicate with the indicated plasmids using Lipofectamine 2000 (Invitrogen). The 293T cells were co-transfected with rat GDNF or rat SHH and U6 promoter driven shRNAs against rat GDNF or rat SHH, or controls (U6 promoter only & shGFP). At 24 hours after treatment, cells were lysed in 100 μl of Cell Disruption Buffer (PARIS kit, Ambion), and 50 μl of the lysate was added to 1 ml of TRIzol for RNA isolation. The remaining lysates were kept for western blot analyses.

Lentiviral vector generation: Feline immunodeficiency virus (FIV) pseudotyped with VSV-G (vesicularstomatitis virus G) were generated by the Gene Transfer Vector Core of the University of Iowa (<http://www.uiowa.edu/~gene/>). Briefly, the shRNA’s were shuttled into FIV vectors as described by Harper et al, 2005 and recombinant FIV particles were produced by using the triple plasmid system. More specifically, the vector plasmid was based on the pVETL backbone containing a GFP reporter gene driven by a cytomegalovirus (CMV) promoter as shown in figure 2E in the main text. The triple transfection system of particle production involves concurrent transfection of 293T cells with three plasmids, followed by harvest of particle-containing culture medium and concentration of particles.

Lentiviral shRNA infection into NSCs*:* FIV viruses carrying the shRNA plasmids and controls were infected into the NSCs in culture at 1MOI for 6 hours. The cells were 80-90% GFP positive at 72 hours post-infection as checked via fluorescence microscopy as well as FACs analysis. A defined portion of cells was transplanted into the animals, and the remaining cells were lysed, RNA and protein collected, and samples analyzed via qRT-PCR and Western Blotting to check knockdown efficiency.

**Immunohistochemistry antibodies**: The concentrations and commercial sources (with catalog numbers) of the primary antibodies used were as follows:

BrdU (1:200, MAB3222), Nestin (1:200, MAB353), RIP (1:500, MAB1580), Tyrosine Hydroxylase (TH, 1:4000, MAB318), Glial Fibrillary Acidic Protein (GFAP, 1:500, S2532) all from *Millipore-Chemicon, Danvers, MA*; Neuronal class III β-tubulin (Tuj1, 1:300, MRB435P) from *Covance, Princeton, NJ*; GDNF (1:500, sc328, for immunohistochemistry), SHH (1:200, sc1194), Doublecortin (Dcx, 1:400, sc8066) from *Santacruz Biotechnology, Santacruz, CA*; hPAP (1:500) from *Accurate, Westbury, NY*; S100β (1:500) from *Sigma, St Louis, MO;* SDF1α (1:250, ab25117) and GDNF (1:500, ab28956, for western blotting) from *Abcam, Cambridge, MA*; Integrin alpha M (CD11b, 1:50, MCA275) and CD68 (1:50, MCA341) from *Serotec, Raleigh, NC*. Detailed descriptions of these antibodies have been provided in our previous publications of Madhavan et al., 2009 and 2012 [5, 9].
